# Extending Trait‐Based Ecology Across Disciplines in the Face of Global Change

**DOI:** 10.1002/ece3.74068

**Published:** 2026-07-25

**Authors:** Thiago Gonçalves‐Souza, Tsun Fung Au, Rose E. Brinkhoff, Morgan R. McPherson, Sarah L. Raubenheimer, Katherine S. Rocci, Yiluan Song, Liting Zheng, Jennifer R. Head, Xinli Chen, Rémi Bardou, Peter B. Reich

**Affiliations:** ^1^ Institute for Global Change Biology, School for Environment and Sustainability University of Michigan Ann Arbor Michigan USA; ^2^ Department of Ecology and Evolutionary Biology University of Michigan Ann Arbor Michigan USA; ^3^ Institute of Arctic and Alpine Research University of Colorado Boulder Colorado USA; ^4^ Michigan Institute for Data Science University of Michigan Ann Arbor Michigan USA; ^5^ Department of Epidemiology, School of Public Health University of Michigan Ann Arbor Michigan USA; ^6^ Department of Renewable Resources University of Alberta Edmonton Alberta Canada; ^7^ Department of Forest Resources University of Minnesota St. Paul Minnesota USA

**Keywords:** cross‐scale integration, global change research, transdisciplinary collaboration

## Abstract

Trait‐based ecology has been successful in linking organismal traits to ecological responses under environmental change and to their effects on ecosystem processes, offering a conceptual foundation with promise for translation across disciplines. Human‐induced environmental changes—climate change, land use alterations, biotic community shifts, and pollution—impact life, physical, health, and socio‐economic systems. Addressing these global change impacts requires an interdisciplinary approach to improve policy and management. However, predicting responses to global changes remains challenging due to cross‐system complexity. We propose a trait‐based approach, encompassing attributes important to the responsiveness of different systems across disciplines as an effective solution to overcome barriers for cross‐system communication. This paper reviews literature on traits central to global change sciences, highlighting their interdisciplinary links. We present examples where a trait‐based approach connects life, physical, social, and health sciences disciplines, spatial scales, and levels of organization. We propose a trait‐based framework for bridging science disciplines by using pathways that connect global change drivers and traits. This framework aims to offer a comprehensive perspective integrating multiple system hierarchies, thereby guiding interdisciplinary research to mitigate environmental threats to society.

## Graphical abstract

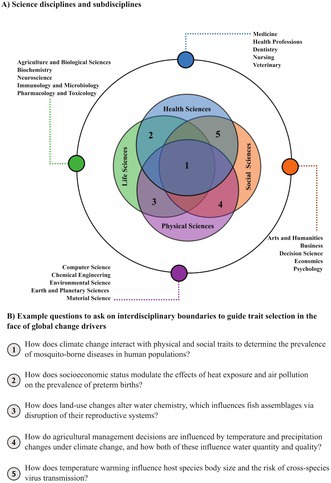

## 1 Introduction

Anthropogenic global changes—inclusive of climate change, land‐use change, biotic community shifts, and pollution—impact social‐ecological systems, causing immediate and downstream effects on life on Earth (Pecl et al. 2017). To address these impacts, many disciplines, including life, health, physical, and social sciences, have been investigating the cascading effects of global change on the Earth system, biodiversity, and people (Bratman et al. 2019; Gilbert and Lynch 2019; Chaudhary et al. 2022; Junker et al. 2023; Romanello et al. 2023). A common but not ubiquitous thread across these disciplines is to focus on the traits (sometimes referred to as attributes, characteristics, or properties; see Glossary, Supporting Information) of organisms or entities, either explicitly or implicitly, to understand how they are affected by or mediate the effects of global change. Traits can be either quantitative (e.g., the diameter of the main and lateral pipes in a municipal sewer system) or qualitative (e.g., whether a highway is made of concrete vs. asphalt). We define traits as well‐defined properties of biotic (e.g., size, chemistry, age, physiological tolerance, disease prevalence, and behavioral adaptability; as in ecological, health, and social sciences) or abiotic entities (e.g., carbon emission policies, and hydrological resilience; as in social and physical sciences), measured at one or multiple scales (Table 1; see Glossary, Supporting Information). Although the term trait has a more established and deep history in ecology (Reich 1987; Reich et al. 1992; Westoby 1998; Violle et al. 2007), where it often refers to measurable organismal properties linked to performance, we use this ecological logic more broadly to identify analogous properties in other disciplines. A trait‐based approach provides a framework for using the traits of organisms, social or physical entities, or systems to confer predictability regarding their responses or changes in the face of global change drivers.

**TABLE 1:** Hierarchy of trait scales for life, health, social, and physical sciences, with equivalent terms aligning across disciplines (with examples in parenthesis).

Trait scale	Life sciences	Health sciences	Social sciences	Physical sciences
Fine scale 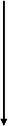	Organism (bird)	Patient (human)	Person/Agent (human)	Physical entity (water column)
	Species population (sparrow population)	Patient cohort/group (workers)	Demographic group (farmers)	Aggregate/collection (lake)
	Species community (different bird species)	Healthcare community (hospital capacity)	Social institution (cooperative)	Network (watershed)
Coarse scale	Ecosystem (tropical forest)	Healthcare system (nationwide health system)	Societal structure (government)	Environmental system (hydrologic system)

*Note:* These scales allow us to identify in which scale a given trait from one specific discipline can have a connection with another discipline, thereby facilitating interdisciplinary assessment of relevant variables to global change research. See additional details in Figure 5.

Trait‐based ecology provides a conceptual foundation for this broader integration across disciplines. In ecology and evolution, traits are used to investigate how global change drivers affect individual performance, biodiversity change and how these changes affect ecosystem processes (Reich 1987; Reich et al. 1992; Wright et al. 2004; Violle et al. 2007; Gonçalves‐Souza et al. 2023). For example, studies use plant traits (e.g., stomatal conductance, wood density, seed mass, height) and animal traits (e.g., body size, dispersal ability) to understand how organisms respond to rising CO_2_, atmospheric warming, drought and ozone pollution (response traits: Reich 1987, Bjorkman et al. 2018, Bruelheide et al. 2018), and how these responses alter ecosystem processes (effect traits: Lundgren et al. 2013; Terrer et al. 2019; Rocci et al. 2021). This response‐and‐effect distinction is one of the central contributions of trait‐based ecology to interdisciplinary translation, because it links attributes both to sensitivity to global change and to consequences for broader system processes. However, to our knowledge, there has not been an assessment of how trait‐based approaches are used across science disciplines. Understanding similarities and differences in trait usage could enhance collaboration across disciplines to mitigate the impacts of global change.

We hypothesize that a trait‐based logic developed in ecology could be readily adapted to other sciences that already often consider how attributes of individuals or entities change in response to global change (see, e.g., Andersson et al. 2021; Fisher et al. 2023), but have not necessarily converged around this as a useful framework. Although disciplines such as social, physical, and health sciences do not explicitly use the word “trait” to assess how global change affects organisms and social or physical entities, they do select relevant characteristics that can be translated into a trait‐based approach. For example, in social sciences, the interaction between humans and biodiversity can be investigated by detecting how human attributes such as emotional, cognitive, and spiritual well‐being are linked with attributes (e.g., colors, sounds, smells, textures) from animals, fungi, and plants (Andersson et al. 2021; Fisher et al. 2023). In physical sciences, stable isotope ratios (e.g., δ^18^ O) of sediments or ice cores are used to reconstruct long‐term climate variability such as warmer or drier periods (Ghosh and Brand 2003; Vystavna et al. 2021). In health sciences, researchers use health outcomes and attributes (e.g., disease state, blood pressure, cholesterol level) to understand the effects of global change drivers on human populations (Giorgini et al. 2017; Gostimirovic et al. 2020) and/or as predictors of disease event risk. Although these disciplines do not use the same terminology, they share both related concepts and a common goal to understand the consequences of global change for biodiversity, people, and systems.

While science disciplines share this common concern, cross‐disciplinary collaboration remains challenging due to differing terminology. Andersson et al. (2021) demonstrated that traits can connect social and life sciences when a trait describes an entity in ways that translate to both ecological functioning and socio‐cultural meaning. The authors exemplified this using plant traits (flower size and shape) that affect nature perception and valuation, which in turn influence societal approval for urban green spaces. To connect these two disciplines, Andersson et al. (2021) established common trait terminology and demonstrated how traits function as relational features across scales and systems. Therefore, a trait‐based approach has potential as a unifying framework for understanding and predicting global change impacts, as previously articulated, but more narrowly, in ecology and related disciplines (e.g., Webb et al. 2010; Andersson et al. 2021; Fisher et al. 2023).

Here, we analyze trait usage in global change research across life, health, physical, and social sciences through literature review and network analysis. We propose a trait‐based framework to unify diverse scientific disciplines, enabling collaborative formulation of research questions to mitigate global change impacts on system processes. This framework links global change drivers with traits, facilitating cross‐scale translations and integration across biological, health, social, and physical systems. To harness traits for addressing global change effects on Earth systems, we provide an operational protocol for translating discipline‐specific trait terminologies by exploring the following research gaps: (1) trait definitions, terminology, and scale harmonization across disciplines; (2) shared versus discipline‐specific traits across scales and systems; and (3) transdisciplinary multiscale integration. This approach identifies traits shared across disciplines that are likely to be extensively applicable across scales, thereby operationalizing cross‐disciplinary trait translation.

## 2 Potential Challenges of Developing a Trait‐Based Common Language Across Science Disciplines

Traits (and analogous terms such as attributes and properties, see Glossary, Supporting Information) are widely used in global change research, but their application within and across science disciplines is fragmented because of terminological ambiguity and scale mismatches, contributing to disciplinary silos. These barriers hinder integration across fields, complicating efforts to address global change research holistically.

### 2.1 Terminological Ambiguity Affects the Use of Shared Traits Across Disciplines

A single trait might be used across disciplines or scales, but sometimes with analogs with different terminology (Keller et al. 2023). For example, while community ecologists explicitly use “body mass” as a trait to study climate change effects on morphological shifts in mammals (Pacifici et al. 2017), health scientists may investigate body mass as a health *outcome* rather than a “trait,” despite both disciplines addressing similar drivers such as warming (Stibel 2023). In addition, the dual roles of traits also cause a terminology challenge as the same trait can be used in different ways, as both a regulator of altered processes and functions (“effect trait,” sometimes called “effect modifier”) and a consequence of global change (“response trait”) (Violle et al. 2007). For example, in the discipline of life science, leaf traits both mediate the effects of global change on plant productivity and soil C accumulation (Chen et al. 2023), while also being altered by elevated CO_2_ and warming (Craine and Reich 2001).

### 2.2 Scales Mismatches Fragment Harmonization Efforts

A single trait such as leaf area can be measured in different ways across scales: individual leaf area in a tree measured manually versus forest canopy cover in a landscape estimated via spectral reflectance (Pacifici et al. 2017). While these measurements can be conceptually linked across scales because they capture related dimensions of resource acquisition, light interception, or storage (Gomarasca et al. 2023), their ecological roles and responses to global change often diverge. For instance, while spectral reflectance in physical science detects changes in atmospheric temperature in response to CO_2_, life sciences link spectral reflectance of a canopy to leaf‐level photosynthetic rates altered by climate and microclimate variations. These differences in drivers and processes create communication silos distancing disciplines when comparing global change drivers and responses across scales (Bruelheide et al. 2018).

These knowledge gaps matter because they hinder transdisciplinary global change research that can be built on theories or models that involve shared traits. On one hand, studies in many disciplines use traits implicitly without identifying them as using a trait‐based approach or adopting the word “trait.” On the other hand, we lack useful methods for researchers to identify related traits from other disciplines, especially when different terms are used for a trait. Therefore, identifying similarities and differences among science disciplines will be fundamental when addressing global change problems across scales and disciplines, and how they affect the link between environment and society.

## 3 Shared Versus Discipline‐Specific Traits Across Scales and System

**FIGURE 1:**
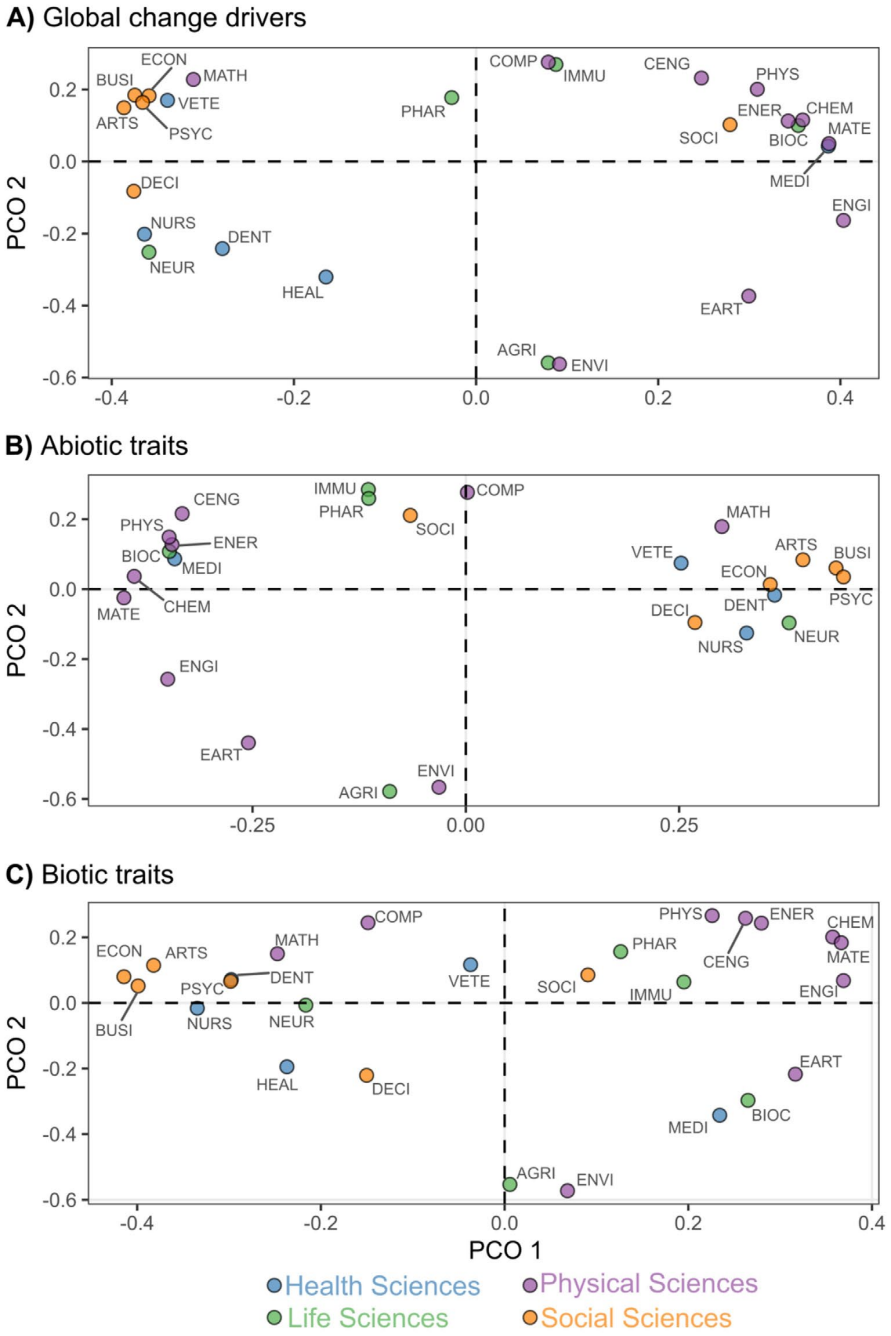
Biplot of a Principal Coordinate Analysis (PCoA) based on a Bray–Curtis dissimilarity index that gives weight to the number of times a specific term (biotic or abiotic traits, or global change drivers) was cited in a subdiscipline (Table S1). Two subdisciplines that are close together in the ordination biplot use similar terms and term frequencies. The color scheme was not included in the analysis and represents just a visual representation of the disciplines. Full names of the subdisciplines included are given in Table S1.

**FIGURE 2:**
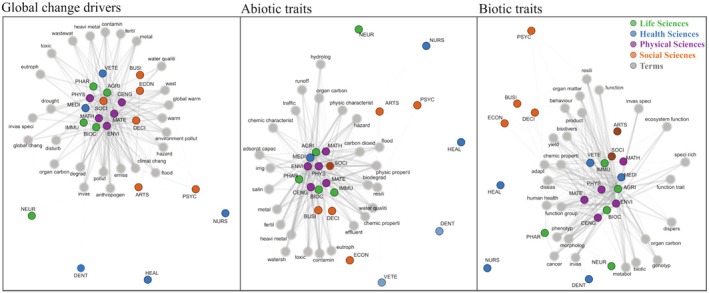
Network graphs exploring the co‐occurrence of abiotic and biotic traits, and global change terms extracted from the title and abstract of the manuscripts. The circles represent nodes that include terms, disciplines, and subdisciplines (based on the ASJC codes, Table S1), and the gray lines indicate the edges that connect nodes. Edge width represents (i) the number of manuscripts that a given term was used in a specific subdiscipline (i.e., edges connecting a subdiscipline with a term), and (ii) the number of times terms were used (term co‐occurrence) in two subdisciplines. Nodes that are closer together suggest a higher frequency of terms used in the same manuscript. Consequently, two subdisciplines positioned in close proximity within the bipartite graph have a large number of manuscripts using similar terms.

A literature search of global change research within the Scopus database (Scopus 2024) followed by a keyword extraction enabled us to identify the use of trait terms (see Section 7) across four broadly defined science disciplines: life sciences, physical sciences, health sciences, and social sciences. We found a marked increase in the number of published articles using trait‐based approaches across all science disciplines over the past two decades, with a total of ~30,000 articles from 1975 to 2024 (Figure S1). We extracted terms related to traits and global change drivers from the titles and abstracts of these articles using a Rapid Automatic Keyword Extract (RAKE) algorithm (Grames et al. 2019), which yielded 7759 terms. Given that this algorithm does not assign predefined meanings to the terms, we manually categorized them as either a biotic trait, an abiotic trait, or a global change driver, in accordance with the definitions provided in the Glossary (Supporting Information). We categorized 1301 terms and excluded 6458 terms that were not related to traits or global change drivers (see Table S2). Examples of categorized terms include “biodegradability,” “irrigated,” “noise pollution,” and “water quality” as abiotic traits, “yield,” “phenotype,” “pathogen,” and “cancer” as biotic traits, and “warming,” “drought,” “flood,” and “pollution” as global change drivers (Figure S2, Table S2). We then used the categorized terms to identify the extent of overlap in the usage of these terms across the four science disciplines through similarity analysis and graphical inspection (Figure S2) and ordination analysis (Figure 1). We also used network analyses to explore the co‐occurrence of terms extracted from the manuscripts (title and abstract) at specific nodes, which represent biotic traits, abiotic traits, global change drivers, and disciplines (Figure 2). By doing this, we were able to identify trait terms shared across disciplines.

**FIGURE 3:**
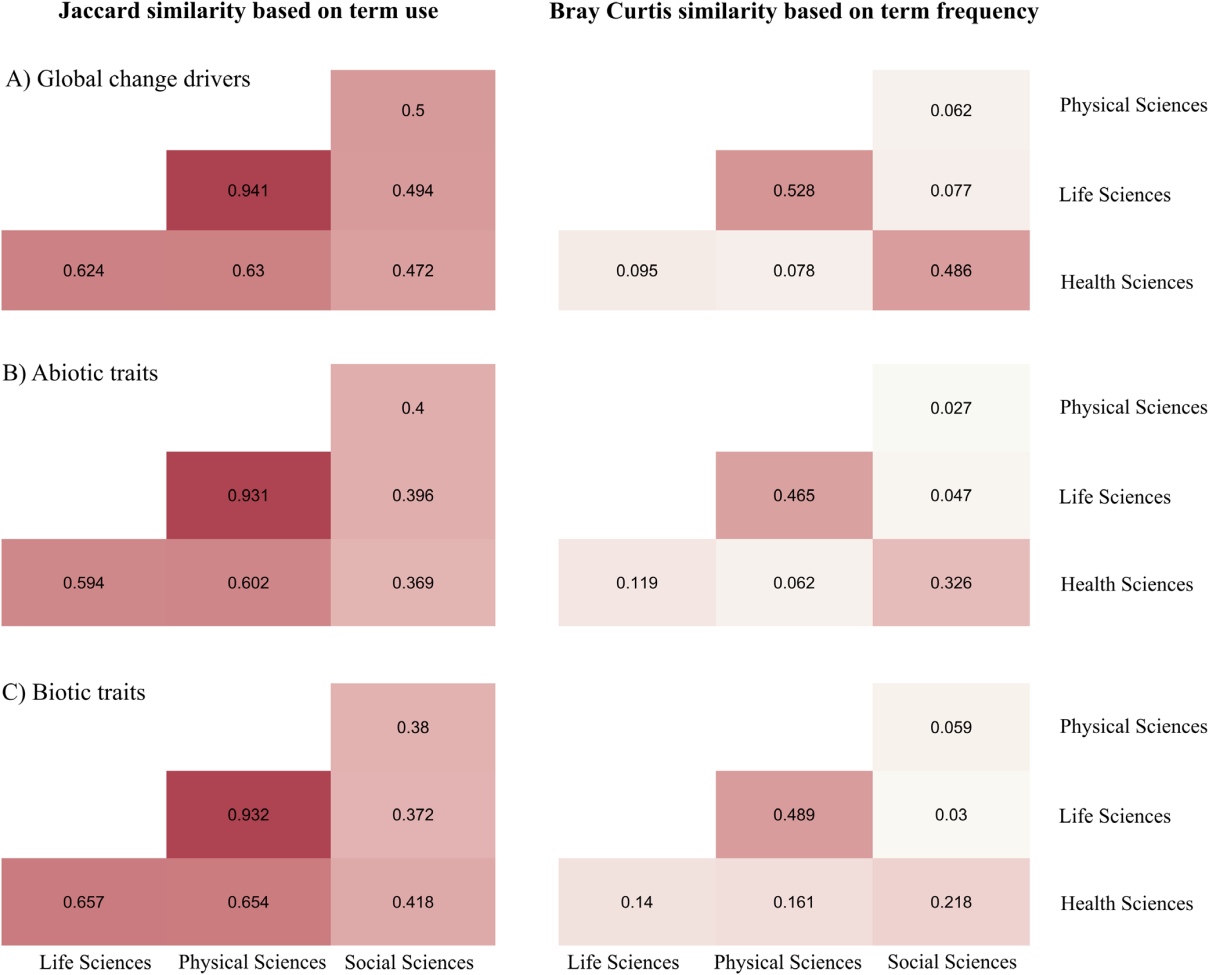
Similarity in the usage of biotic trait terms represented by the Bray‐Curtis index, which considers the frequency of term use between disciplines. The Jaccard index, in turn, compares the similarity in term use without considering use frequency. The values of both indices vary between 0 (completely different trait use or trait frequency use) to 1 (completely similar trait use or trait frequency use).

Among global change literature that involves traits across disciplines, “climate change,” “pollution,” “invasive species,” and “drought” were the most common global change drivers, while “functional traits,” “invasive,” “productivity,” and “adaptation” were the most common biotic trait terms, and “contaminants” and “heavy metals” were the most common abiotic traits (Figure S2). These trait terms have considerable variability in specificity (e.g., physical properties vs. toxicity), with the most common terms representing broad categories. The most significant variations in trait terms between disciplines relate not to the content—that is, how similar the terms are—but rather to the frequency with which the different terms are used (Figure 3). While all pairs of disciplines share a substantial number of terms, this similarity drops considerably when frequency is taken into account. Of the four sciences examined, social sciences shared the least similarities in trait and global change driver term usage when comparing either term use or term frequency, while life and physical sciences share the most (Figure 3). Moreover, we also compared the similarity in frequencies across subdisciplines (Table S3). We found that even subdisciplines from distinct disciplines shared similar term use and frequency (Figure 1). For example, Agricultural and Biological Sciences (AGRI) from life sciences and Environmental Science (ENVI) from physical sciences used very similar terms (Figure 1). Likewise, Dentistry (DENT, health sciences) shared similar terms with Neuroscience (NEUR, life sciences). Notably, but perhaps not surprisingly, disciplines that overlapped the most in biotic trait use frequency also overlapped most strongly in abiotic traits and global change drivers (Table S3), which can facilitate the connections of these disciplines or subdisciplines (Figures 1 and 3).

We identified a core set of terms that form a cluster connecting at least one subdiscipline from each discipline (Figure 2). These clusters include nearly all subdisciplines from life and physical sciences across all terms. By quantifying the linkage between traits and disciplines, we were able to identify which subdisciplines share similar language regarding traits, even when they belong to different disciplines. For example, AGRI from life sciences, ENVI from physical sciences, Medicine (MEDI) from health sciences, and SOCI from social sciences show shared usage of chemical and physical characteristics as abiotic traits, and of functional traits and ecosystem functioning as biotic traits. This commonality could be used to bridge gaps between these disciplines. However, subdisciplines within the health and social sciences tended to remain more peripheral within these networks. This finding suggests that the terminologies used in the latter two disciplines differ from the core list that interconnects life and physical sciences (Figure 2). Unlike life and physical sciences, health and social sciences rarely use the term “trait” to describe these characteristics, opting instead for “outcomes” or “properties.” Therefore, it is crucial to recognize the distinct terms used across disciplines to foster effective interdisciplinary connections.

We found considerable overlap in terms of traits and drivers of global change across science disciplines. However, bridging some disciplines, especially within the health sciences (e.g., Veterinary) will pose a significant challenge (see Figures 1 and 2). Nevertheless, the overall generally high levels of similarities between disciplines in their use of traits (Figures 1 and 3) to understand global change effects suggest there is an enormous potential to connect disciplines even in very different areas.

## 4 Developing a Multi‐Scale and Multi‐Discipline Trait‐Based Framework

**FIGURE 4:**
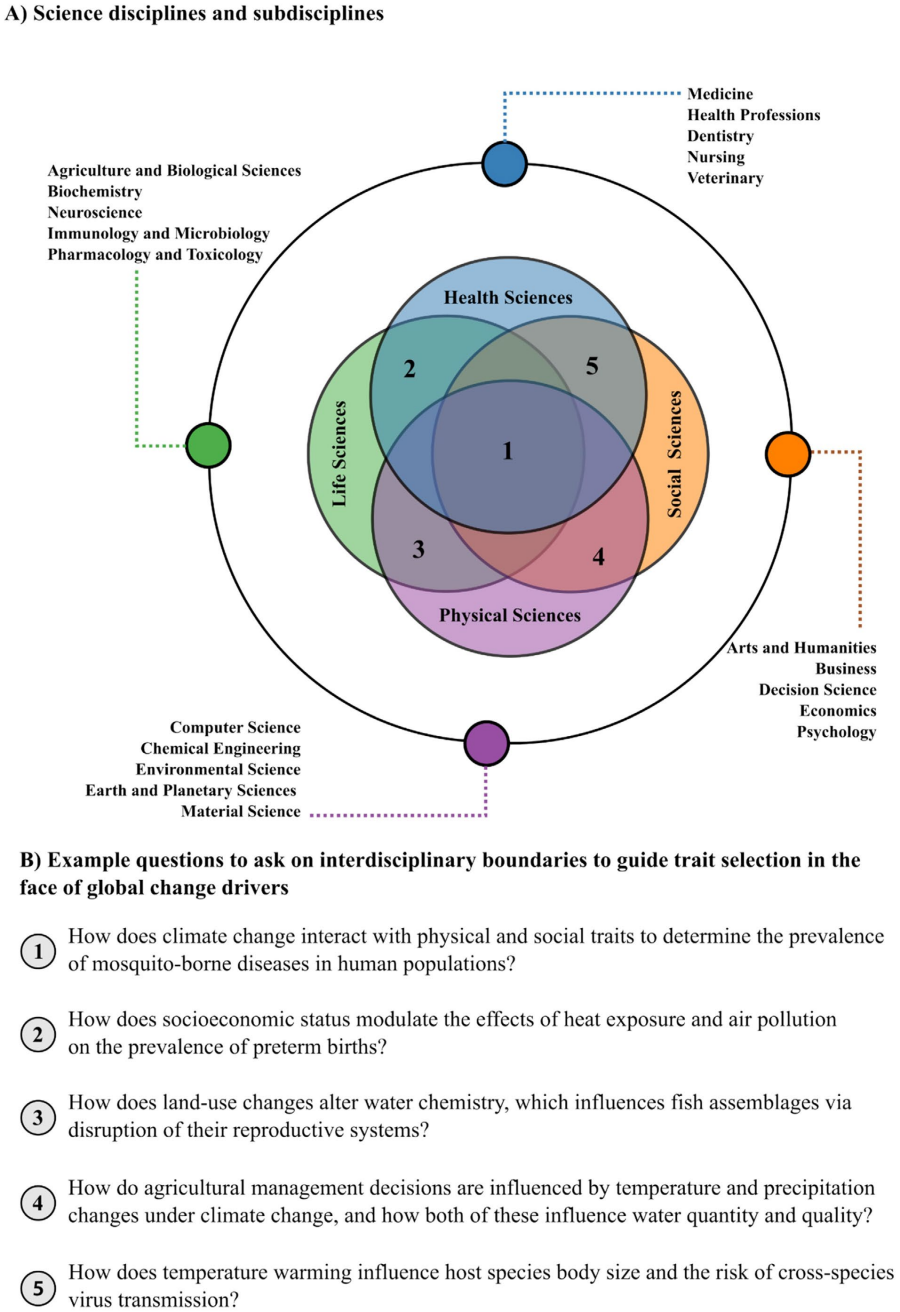
(A) Venn diagram showing science disciplines and subdisciplines. The shared information (overlap between one or multiple disciplines) includes processes, scales, global change drivers, and trait terms that are commonly used in two or more disciplines. The proposed trait‐based framework uses this common language to integrate them by recognizing cross‐discipline similarities or interdisciplinary boundaries. (B) We provide some example questions (and developed some in the main text) that require interdisciplinary knowledge to advance our understanding of the effects of global change on Earth systems.

**FIGURE 5:**
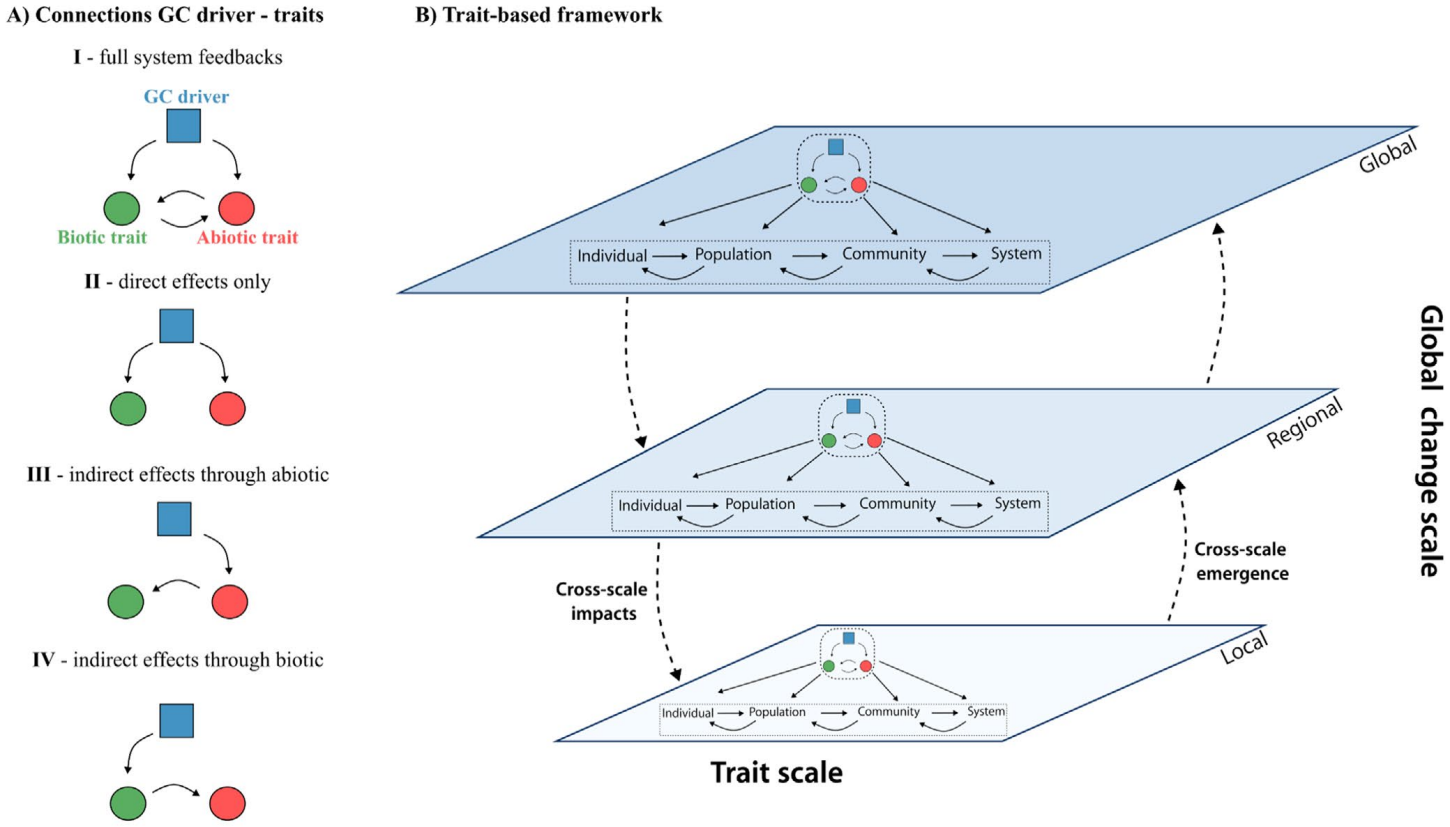
(A) Potential scenarios connecting global change drivers (GC drivers, blue squares) with traits are described as follows: Scenario I shows full system feedbacks, where a GC driver can independently affect biotic (green circles) and abiotic (red circles) traits, which then influence each other. Scenario II demonstrates direct effects only, with no interactions between trais. Scenario III illustrates how a GC driver can impact abiotic traits, which subsequently influence biotic traits (indirect effects). Scenario IV represents the reverse of indirect effects, where GC drivers impact biotic traits, which then affect abiotic traits. (B) These connections form the central part of the proposed trait‐based framework, which crosses disciplines and allows for an explicit organization of trait and global change scales based on a macrosystems perspective (Heffernan et al. 2014). Trait scale typically varies from individual (e.g., an individual bird in life sciences or a person in health or social sciences) to population, community, and (eco)systems (see Table 1 for term use across disciplines). The global change scale ranges from local to global, and within each scale, these GC drivers can affect traits at different trait scales. Furthermore, changes at the local scale can reverberate across different trait scales (from individuals to systems), which, in turn, can emerge at larger scales by affecting other biotic and abiotic traits and altering processes across multiple scales. Conversely, global change processes acting at global or regional scales can cascade down to the local scale, affecting the interaction between traits (biotic and abiotic) and trait scales.

Here, we propose a trait‐based framework that bridges life, health, social, and physical sciences (Figures 4A and 5), adopting a macrosystems perspective used in ecology (Heffernan et al. 2014). This framework directly addresses the knowledge gaps identified in this study. First, it identifies shared traits across disciplines. Second, it provides terminology for categorizing traits as both abiotic and biotic, linking them to global change drivers. Importantly, the inclusion of abiotic traits allows for the integration of disciplines such as physical sciences into trait‐based global change research. We use trait‐based ecology as the starting template for this framework because it already links environmental change drivers, traits, and ecosystem functioning across scales.

The first step in using the framework is to identify the focal traits and global change drivers (hereafter GC drivers) pertinent to the question, and then the scales at which abiotic and biotic traits, as well as the GC drivers, vary and affect each other (Figure 5A,B). For instance, in life and health sciences, GC drivers can influence traits across the “trait scale,” at the individual level (organism or patient), the population level (species population or patient cohort), the community level (species or healthcare community), or the system level (ecosystem or healthcare system) (Table 1 and Figure 5B, *x*‐axis). Thus, connections between these disciplines through the trait scale are facilitated by identifying the relevant entities from the finest scale (individual) to the largest scale (system, Table 1). Regardless of the discipline, when GC drivers cause trait changes at the individual level, they can also affect the whole system level, and vice versa (Figure 5B, inset symbols, and solid arrows connecting trait levels).

In addition to the trait scale, the framework represents different spatial scales in which GC drivers and traits are collected, which varies from local to regional and global (Figure 5B, global change across spatial scales on the *y*‐axis). Processes that occur at larger spatial scales can impact processes at finer spatial scales (Figure 5B, cross‐scale impacts), and processes at finer scales emerge as broader patterns at larger spatial scales (Figure 5B, cross‐scale emergence).

## 5 Integrating Traits and Scales Across Disciplines for Addressing Global Change Research

To demonstrate the usefulness of the proposed trait‐based framework, we highlight examples of five interdisciplinary study systems that vary in complexity (i.e., number and types of disciplines integrated) and scale (i.e., different trait and global change scales). The first example describes a four‐way interaction of social, health, physical, and life sciences, and the other four represent paired connections between two disciplines (Figure 4A). To be able to harmonize the terminology and scales, we need to identify the GC drivers and traits involved, whether they are biotic or abiotic, and the relevant science discipline they come from. To do that, we identified relevant variables, disciplines, and scales in the examples below.

### 5.1 How Does Climate Change Interact With Physical and Social Traits to Determine the Prevalence of Mosquito‐Borne Diseases in Human Populations (Figure 4B, Question 1)?

This example illustrates how integrating trait‐based frameworks across biological, physical, and social sciences can clarify the complex drivers of mosquito‐borne disease dynamics under climate change. By explicitly identifying and linking traits across disciplines, we can better understand and address such multifaceted global change challenges.

Mosquitoes transmit a range of pathogens—including those causing malaria, dengue, yellow fever, and Japanese encephalitis—with some pathogens more likely to infect specific mosquito genera (Aedes, Culex, Anopheles) (biotic traits, health sciences). These vector species differ in their physiological responses to environmental conditions, particularly temperature and rainfall (Caminade et al. 2014), which shape their fecundity, development, and survival (biotic traits, life sciences). Such climatic factors (GC drivers) are especially influential in the tropics, where mosquito‐borne diseases are most common.

Climate change—including rising temperatures, altered rainfall, and extreme events like El Niño (GC drivers)—can shift mosquito distributions (biotic trait, life sciences) poleward (Martens et al. 1999), increasing disease risks (biotic trait, health sciences) in temperate regions (Pecl et al. 2017). For example, in 2023, dengue cases reached historic levels: ~5 million reported cases and over 5000 deaths across 80+ countries (WHO – World Health Organization 2023a). This spike coincided with the 2023 El Niño, marked by elevated temperature, precipitation, and humidity (GC drivers). While 80% of cases occurred in the Americas, three European countries (Italy, France, Spain) reported 128 locally transmitted (autochthonous) cases—outside dengue's usual endemic range (WHO – World Health Organization 2023a).

The distribution and impact of mosquito‐borne diseases are also shaped by interacting physical and social factors. Standing water provides egg‐laying sites, while water quality—determined by pH, oxygen levels, and eutrophication (abiotic trait, physical sciences)—affects larval survival differently across mosquito species (Avramov et al. 2024). Urban environments (abiotic trait, physical sciences) influence mosquito abundance through water pollution and also affect biting behavior (biotic trait, life sciences), given species‐specific differences in activity timing, host‐seeking range, and resting preferences (Gubler 2011; Baker et al. 2022).

Social and economic factors and health infrastructure further mediate disease risk by influencing preparedness and response capacity. These include access to healthcare, surveillance systems, and vector control (abiotic traits, health sciences), along with sanitation infrastructure and international travel and trade networks (abiotic traits, social sciences) that can facilitate mosquito spread (Gubler 2011; Baker et al. 2022). The use of a trait‐based framework would help to disentangle the biological, physical, and socioeconomic drivers of mosquito‐borne disease risk. This example extends the ecological understanding that physiological, behavioral, and life‐history traits mediate species responses to climate into a health‐risk framework. Especially for biological diseases, the shared ecological context should enable closer coordination and shared trait approach between health and ecological sciences.

### 5.2 How Does Temperature Warming Influence Pathogen Thermotolerance and Subsequent Virulence in Host Infection and Drug Resistance? (Figure 4B, Question 2)?

This example bridges health and life sciences to show how global change drivers like temperature warming affect pathogen biology and human health. Warming (GC driver) alters pathogen life cycles and increases virulence (biotic trait, life sciences), including in environmental human‐pathogenic fungi (Gusa et al. 2020). Infections by fungal pathogens have risen sharply, especially in immunocompromised individuals and individuals living with HIV (Chen et al. 2017), heightening the need for new treatments (Garcia‐Solache and Casadevall 2010).

Environmental fungal pathogens such as *Cryptococcus neoformans* must rapidly adapt to survive under host‐associated heat stress. These adaptations include genomic mutations linked to increased virulence and antifungal drug resistance (biotic traits, health sciences) (Gusa et al. 2020). The public health impact is especially severe in regions like sub‐Saharan Africa, where HIV/AIDS‐related cryptococcal meningitis causes high mortality (Rajasingham et al. 2017). Warmer temperatures in these areas are also linked to increased male migration and sex‐market use, contributing to HIV spread (biotic traits, social sciences) (Baker 2020).

These trends highlight the urgent need for coordinated research across disciplines to address environment‐to‐host transmission risks under global heat stress. Given that thermotolerance responses vary across environmental pathogens—from bacteria to fungi (biotic traits, health sciences)—people with HIV are more susceptible to pathogens (biotic trait, health sciences), and HIV spread is related to shifting social dynamics (biotic trait, social sciences); a trait‐based framework is essential for predicting future virulence and drug resistance potential.

### 5.3 How Do Land‐Use Changes Alter Water Chemistry, Which Influences Fish Assemblages via Disruption of Their Reproductive Systems (Figure 4B, Question 3)?

This example integrates physical and life sciences to illustrate how global change drivers—specifically forest loss and agriculture (GC drivers)—affect both fish reproductive traits (biotic traits, life sciences) and water chemistry (abiotic traits, physical sciences). The removal of riparian forests alters fish assemblages, leading to declines in some species and increases in others (Jones III et al. 1999). These shifts trigger cascading ecological effects, including changes in microhabitat use, swimming strategies, and behavior—some driven by endocrine‐disrupting chemicals from agricultural runoff (abiotic traits, physical sciences; biotic traits, life sciences) (Jones III et al. 1999).

Habitat degradation and chemical pollution further alter fish feeding behavior and favor sediment‐tolerant species, as deforestation increases fine sediment deposits in riffles (Lobón‐Cerviá et al. 2016). These changes often facilitate the spread of introduced species, reduce native habitat availability, and modify trophic structures (biotic traits, life sciences). In the Amazon River floodplain, forest cover has been shown to support higher fish diversity, while deforestation leads to spatial homogenization of assemblages (Arantes et al. 2018).

In sum, land‐use change disrupts aquatic systems by altering water quality, species composition, and key reproductive and behavioral traits in fish. This case highlights the importance of linking physical and biological traits to understand the complex consequences of land‐use change on freshwater biodiversity.

### 5.4 How Are Agricultural Management Decisions Influenced by Temperature and Precipitation Changes Under Climate Change, and How Do Both of These Influence Water Quantity and Quality (Figure 4B, Question 4)?

This example integrates physical and social sciences to illustrate how climate change affects agricultural decisions and aquatic systems. Almeida et al. (2018) modeled a Portuguese river under various climate and socio‐economic scenarios. In one scenario, high economic growth coupled with low environmental awareness (biotic traits, social sciences) led to increased atmospheric CO_2_ (GC driver), rising regional temperatures (GC driver), and reduced precipitation (GC driver). The decreased precipitation reduced surface runoff and river flow (abiotic traits, physical sciences), prompting farmers to increase irrigation (biotic traits, social sciences), which further diminished flow. Lower river flow concentrated nutrients such as phosphorus and nitrogen (abiotic traits, physical sciences), with nitrogen levels intensified by greater fertilizer use linked to low environmental awareness (biotic traits, social sciences). The authors also demonstrated that at the regional scale, these changes led to increased chlorophyll *a* (biotic trait, life science) at the river outlet—an indicator of elevated phytoplankton biomass due to high nutrient levels (abiotic traits, physical sciences). This algal growth reduced dissolved oxygen (abiotic trait, physical sciences) through decomposition (biotic traits, life sciences), resulting in fish die‐offs and declining food security for local communities. The resulting economic strain (biotic traits, social sciences) at the local level fed back to regional and global scales.

This example demonstrates how climate change drivers interact with social behavior and biogeophysical processes across scales. By applying trait‐based reasoning, we can trace the cascading effects of climate–society–ecosystem interactions from global CO_2_ emissions down to local water quality and livelihoods.

### 5.5 How Does Socioeconomic Status Modulate the Effects of Heat Exposure and Air Pollution on the Prevalence of Preterm Births (Figure 4B, Question 5)?

This Example Integrates Health and Social Sciences, Highlighting How Individual and Neighborhood‐Level Traits Mediate the Effects of Environmental Stressors on Human Health—An Important Inclusion Given the Peripheral Role of Social Sciences in Earlier Network Analyses (Figure 3). Preterm Birth—Defined as Birth Before 37 Weeks of Gestation (Biotic Trait, Health Sciences; WHO – World Health Organization 2023b)—is a major contributor to child mortality, associated with 1 million deaths globally each year due to respiratory complications and infection risk (Chawla and Agarwal 2022).

Exposure to extreme heat (GC driver) increases the risk of preterm birth. A systematic review found that four of five studies reported a positive association, with a median estimate of a 15% increase in risk (Bekkar et al. 2020). Heat stress may induce labor through hormonal and uterine changes (Dreiling et al. 1991) and pregnant individuals may be physiologically more susceptible due to increased weight and metabolic rate (Marie et al. 2005).

Socioeconomic status (abiotic trait, social sciences) and race/ethnicity (biotic trait, social sciences) further shape vulnerability. Globally, 65% of preterm births in 2020 occurred in sub‐Saharan Africa and South Asia (Ohuma et al. 2023). In the U.S., African American individuals experienced a 50% higher rate of preterm birth than White individuals in 2022 (CDC – Centers for Disease Control and Prevention 2023). These disparities are often linked to neighborhood‐level traits such as unequal access to healthcare and disproportionate exposure to heat and pollution. For example, tree canopy cover (a biotic trait) is lower in racially segregated, economically deprived neighborhoods, contributing to greater urban heat exposure (Locke et al. 2021). Access to antenatal care—protective against preterm birth—is also lower among low‐income and minority populations (Hollowell et al. 2011).

Consequently, the combined effects of heat, air pollution, and social disadvantage intensify risk, with stronger associations found among Black mothers compared to White mothers (Bekkar et al. 2020). This example underscores how trait‐based reasoning across disciplines can illuminate the mechanisms by which global change drivers exacerbate health inequities.

## 6 Conclusions

Rather than narrowing trait‐based research to organismal ecology alone, we argue that ecology provides a well‐established conceptual foundation from which trait‐based reasoning can be translated to other disciplines confronting global change questions. Our study shows this trait‐based approach, already used in part and described with different terminology among different sciences, could be leveraged to enable better communication and interdisciplinary connections among disparate fields. For example, our analyses revealed that life and physical sciences share core trait terms (e.g., chemical properties), validating the feasibility of an integrative framework between these disciplines. However, disciplines such as social and health sciences have subdisciplines that are more peripheral with respect to trait language, emphasizing the need for terminology exchange between disciplines. The proposed trait‐based framework advances our ability to bridge scientific disciplines and develop interdisciplinary global change research. This framework not only bridges disciplines but also enables researchers to investigate the relationship between global change drivers and traits across scales—both spatial (e.g., from local to global) and hierarchical (e.g., organizational levels)—ranging from individual organisms or units to entire systems. We offer examples illustrating the potential for interdisciplinary integration, even among fields with limited overlap in terms of global change drivers and traits, such as the social sciences. Moreover, this framework provides guidelines for policymakers to design interventions across disciplines and scales to address the effects of global change drivers, such as climate change, on the interaction between environment and society. Indeed, the challenges posed by our rapidly changing planet are multifaceted and complex, impacting biological, health, social, and physical systems. Addressing the myriad consequences of global change calls for intricate solutions, which require expansive collaborative research that transcends the boundaries of individual disciplines.

## 7 Methods

To contextualize and illustrate the use of the trait concept relative to global environmental change in different disciplines, we use a modified version of a systematic review. Systematic reviews are commonly used to highlight key knowledge capacity, gaps, and issues in specific scientific fields (Berrang‐Ford et al. 2015; Acevedo et al. 2020; Moore et al. 2021), but are less often used across disciplines. Additionally, there are trade‐offs between large surveys of published literature and more comprehensive but narrower systematic reviews that not only query the literature but analyze how well papers match. Herein we have used the “front‐end” of systematic reviews—a systematic and transparent data collection protocol—but a limited (rather than comprehensive) examination of the collected papers, due to their large number. Instead, we extracted keywords in two approaches. We first retrieved keywords provided by authors and identified by Scopus using the following search terms: TITLE‐ABS‐KEY((trait OR attribute OR “functional group” OR “functional guild”) OR ((health W/2 propert*) OR (health W/2 character*) OR (social W/2 propert*) OR (social W/2 character*) OR (physical W/2 propert*) OR (physical W/2 character*) OR (abiotic W/2 propert*) OR (abiotic W/2 character*) OR (biotic W/2 propert*) OR (biotic W/2 character*)) AND (“climat* change” OR “global environmental change” OR “global change” OR “climat* warming” OR “global warming” OR “anthropogenic impact” OR eutrophication OR “precipitation change” OR “land use change” OR “land‐use change” OR “land cover change” OR “fire regime change” OR invasion OR “emerging disease” OR “biodiversity loss” OR pollution OR urbanization OR “sea‐level rise” OR “sea level rise” OR “increas flood*” OR “increas* storm*” OR “increas* heat wave*” OR “increas* CO2” OR “increas* ozone”)). We also used the All Science Journal Classification Codes (ASJC) and the four Scopus science disciplines as “subject areas” (Table S1): health, life, physical, and social sciences. The ASJC codes also include subdisciplines within each subject area (Table S1). The keywords were then combined with the ASJC codes (subject areas and subdisciplines) to construct a manuscript search that targeted those codes so we can compare (dis)similarities within and between science disciplines.

We then expanded the set of keywords to include terms related to trait and global change drivers that were not represented in the author and Scopus‐provided keywords (Table S2). We extracted keywords from the titles and abstracts using a simplified version of the rapid automatic keyword extract (RAKE) algorithm in R package *litsearchr* (Grames et al. 2019). RAKE is an unsupervised method that uses prescribed stopwords and phrase delimiters as well as document‐specific word co‐occurrence graphs to detect the most relevant words or phrases (Rose et al. 2010). Specifically, we extracted multi‐word keywords that were no longer than five words and were detected in at least two articles. Keywords extracted from both approaches were combined for the subsequent processing and analyses.

We performed the following preprocessing steps for the extracted keywords. We determined the word stems of all keywords with the R package *tm* (Feinerer et al. 2008) to combine those with the same word stems. Keywords with the same stems (e.g., “functional group” and “functional groups”) were replaced with the version with a higher occurrence. We retained the top 3000 keywords for manual labeling. We first screened out irrelevant keywords (e.g., “springer”), then we labeled if a keyword is a synonym or specific term of “trait” (referred to as “trait terms”) or global change driver (referred to as “global change terms”) (more details in Text S1).

### 7.1 Network Analysis

We performed bipartite network analyses to visualize the associations between trait terms and disciplines, as well as the associations between trait terms and global change drivers. Bipartite networks are a special type of network where nodes are of two distinct types or sets, so that edges (connections) only exist among nodes of the different sets (Junker et al. 2023). They are often used to analyze text data to provide insights into relationships between documents and vocabulary (Li et al. 2016; Wang et al. 2018).

To understand how trait terms are used in articles from different disciplines, we summarized trait terms by subdisciplines, counting the number of occurrences in each subdiscipline. Subdisciplines and trait terms made two types of nodes, with subdiscipline nodes color‐coded by the four general disciplines (life, physical, health, and social sciences). Edges connected subdisciplines to trait terms, weighted by the number of occurrences. In this bipartite network, subdisciplines clustered together when they shared similar trait terms, and trait terms clustered together when they occurred in similar subdisciplines.

In order to understand how trait terms are used to address global change drivers, we summarized pairs of trait terms and global change terms that co‐occurred in articles, counting the number of co‐occurrences. Trait terms and global change terms were used as categorical terms to make two types of nodes. Edges connected trait terms to global change terms, weighted by the number of co‐occurrences. In this bipartite network, trait terms clustered together when they co‐occurred with similar global change terms, and *vice versa*. Networks were visualized with the R packages *igraph* (Csardi and Nepusz 2006) and *GGally* (Schloerke et al. 2024).

## Author Contributions

**Thiago Gonçalves‐Souza:** conceptualization (equal), data curation (lead), formal analysis (lead), visualization (lead), writing – original draft (lead), writing – review and editing (lead). **Tsun Fung Au:** conceptualization (equal), formal analysis (equal), methodology (equal), visualization (equal), writing – original draft (equal), writing – review and editing (equal). **Rose E. Brinkhoff:** conceptualization (equal), data curation (equal), methodology (equal), visualization (equal), writing – original draft (equal), writing – review and editing (equal). **Morgan R. McPherson:** methodology (equal), visualization (equal), writing – original draft (equal), writing – review and editing (equal). **Sarah L. Raubenheimer:** conceptualization (equal), formal analysis (equal), methodology (equal), visualization (equal), writing – original draft (equal), writing – review and editing (equal). **Katherine S. Rocci:** conceptualization (equal), methodology (equal), visualization (equal), writing – original draft (equal), writing – review and editing (equal). **Yiluan Song:** conceptualization (equal), data curation (lead), formal analysis (lead), methodology (equal), visualization (equal), writing – original draft (equal), writing – review and editing (equal). **Liting Zheng:** conceptualization (equal), methodology (equal), visualization (equal), writing – original draft (equal), writing – review and editing (equal). **Jennifer R. Head:** visualization (equal), writing – original draft (equal), writing – review and editing (equal). **Xinli Chen:** visualization (equal), writing – review and editing (equal). **Rémi Bardou:** conceptualization (equal), visualization (equal), writing – review and editing (equal). **Peter B. Reich:** conceptualization (lead), funding acquisition (lead), writing – original draft (equal), writing – review and editing (equal).

## Funding

This work was supported by the Institute for Global Change Biology and National Science Foundation Grant NSF‐DBI‐2021898.

## Conflicts of Interest

The authors declare no conflicts of interest.

## Supporting information


**Table S1:** List of disciplines, subdisciplines, and codes.
**Text S1:** Term classification into global change drivers, biotic and abiotic traits.
**Table S3:** Correlation of the Procrustes rotation comparing the scores of the Principal Coordinate Analysis comparing term usage in the different disciplines/subdisciplines.
**Figure S1:** (A) Number and (B) cumulative proportion of scholarly publications from different science disciplines that include keywords related with traits (and similar terms) and global change drivers.
**Figure S2:** The most common terms used in the four science disciplines. (A) global change drivers, (B) abiotic traits, and (C) biotic traits. Filled circles represent the top 10 most cited terms in a given discipline, while non‐filled circles represent terms that do exist in a given discipline, but are not the most common. The terms in the *y*‐axis were organized (from top to bottom) in descending order based on the average number of papers across disciplines.

**Table S2:** List of terms and manual categorization.

## Data Availability

All data supporting the findings of this study are available in the manuscript and its Table S2. Additional materials (including code) are available from the corresponding author upon reasonable request.
