# Supplementary material for: Extending Trait‐Based Ecology Across Disciplines in the Face of Global Change
Source: Ecol Evol. 2026 Jul 25;16(7):e74068. doi: 10.1002/ece3.74068 (PMC13401212; doi:10.1002/ece3.74068)
Supplement: Supplementary file 1 — Table S1: List of disciplines, subdisciplines, and codes. Text S1: Term classification into global change drivers, biotic and abiotic traits. Table S3: Correlation of the Procrustes rotation comparing the scores of the Principal Coordinate Analysis comparing term usage in the different disciplines/subdisciplines. Figure S1: (A) Number and (B) cumulative proportion of scholarly publications from different science disciplines that include keywords related with traits (and similar terms) and global change drivers. Figure S2: The most common terms used in the four science disciplines. (A) global change drivers, (B) abiotic traits, and (C) biotic traits. Filled circles represent the top 10 most cited terms in a given discipline, while non‐filled circles represent terms that do exist in a given discipline, but are not the most common. The terms in the y‐axis were organized (from top to bottom) in descending order based on the average number of papers across disciplines. [file ECE3-16-e74068-s002.docx]

**Supplementary Material**

**EXTENDING TRAIT-BASED ECOLOGY ACROSS DISCIPLINES IN THE FACE OF GLOBAL CHANGE**

Thiago Gonçalves-Souza, Tsun Fung Au, Rose E. Brinkhoff, Morgan R. McPherson, Sarah L. Raubenheimer, Katherine S. Rocci, Yiluan Song, Liting Zheng, Jennifer R. Head, Xinli Chen, Rémi Bardou & Peter B. Reich

**Content:**

**Glossary**

**Table S1.** List of disciplines, subdisciplines, and codes

**Supplementary text 1.** Term classification into global change drivers, biotic and abiotic traits.

**Table S2.** List of terms and manual categorization (file attached in the system).

**Table S3.** Correlation of the Procrustes rotation comparing the scores of the Principal Coordinate Analysis comparing term usage in the different disciplines / subdisciplines.

**Figure S1.** (A) Number and (B) cumulative proportion of scholarly publications from different science disciplines that include keywords related with traits (and similar terms) and global change drivers.

**Figure S2.** The most common terms used in the four science disciplines. A) global change drivers, B) abiotic traits, and C) biotic traits. Filled circles represent the top 10 most cited terms in a given discipline, while non-filled circles represent terms that do exist in a given discipline, but are not the most common. The terms in the y-axis were organized (from top to bottom) in descending order based on the average number of papers across disciplines.

| **Glossary**  **Global change:** large-scale, human-driven changes that modify Earth’s physical, biological, and social processes; for our purposes these include climate change (atmospheric warming, elevated atmospheric carbon dioxide, altered precipitation patterns, climate extremes, and wildfire), pollution (nutrients, metals, synthetic and artificial compounds), land use change (agricultural management, urbanization, deforestation, habitat loss), and biotic change (biodiversity loss, biotic invasion).  **Traits:** a well-defined property of biotic (ecological, health, and social sciences) or abiotic entities (social, physical sciences), measured at one or multiple scales (Table 1). These are often used as a proxy for some aspect of system properties to help compare within and across species/systems. Traits can be either quantitative (e.g., the diameter of the main and lateral pipes in a municipal sewer system) or qualitative (e.g., whether a highway is made of concrete vs. asphalt). These are also called attributes, characteristics, or properties depending on the discipline.  **Trait-based approach for global change research:** any method that uses traits (or synonymous terms such as attributes, characteristics, or properties) to confer predictability regarding the responses or changes of entities across scales (Table 1) in the face of global change drivers.  **Functional trait:** any abiotic or biotic trait that impacts system functions or processes (functional traits can be effect and/or response traits as well).  **Effect trait:** any trait that affects system functions or processes.  **Response trait:** any trait that is affected by a global change driver and that may also be associated with the system’s responses.  **Scales(s):** the spatial, or temporal, or hierarchical lens from which responses are viewed (Table 1, main text). |
| --- |

**Table S1**. List of disciplines, subdisciplines, and codes as defined by Scopus, which group science subdisciplines in the “All Science Journal Classification Codes (ASJC)” for the four disciplines: health, life, physical, and social sciences. This classification is used by Scopus to categorize journals and scholarly publications into these disciplines. More information: <https://service.elsevier.com/app/answers/detail/a_id/12007/supporthub/scopus/>

| **Discipline** | **Subdiscipline** | **Code** |
| --- | --- | --- |
| Health sciences | Dentistry | DENT |
| Health sciences | Health Professions | HEAL |
| Health sciences | Medicine | MEDI |
| Health sciences | Nursing | NURS |
| Health sciences | Veterinary | VETE |
| Life sciences | Agricultural and Biological Sciences | AGRI |
| Life sciences | Biochemistry, Genetics, and Molecular Biology | BIOC |
| Life sciences | Immunology and Microbiology | IMMU |
| Life sciences | Neuroscience | NEUR |
| Life sciences | Pharmacology, Toxicology, and Pharmaceutics | PHAR |
| Physical sciences | Chemical Engineering | CENG |
| Physical sciences | Chemistry | CHEM |
| Physical sciences | Computer Science | COMP |
| Physical sciences | Earth and Planetary Sciences | EART |
| Physical sciences | Energy | ENER |
| Physical sciences | Engineering | ENGI |
| Physical sciences | Environmental Science | ENVI |
| Physical sciences | Material Science | MATE |
| Physical sciences | Mathematics | MATH |
| Physical sciences | Physics and Astronomy | PHYS |
| Social sciences | Arts and Humanities | ARTS |
| Social sciences | Business, Management, and Accounting | BUSI |
| Social sciences | Decision Sciences | DECI |
| Social sciences | Economics, Econometrics, and Finance | ECON |
| Social sciences | Psychology | PSYC |
| Social sciences | Social sciences | SOCI |

**Supplementary Text 1.** Term classification into global change drivers, biotic and abiotic traits.

We explained in the methods section that we used the rapid automatic keyword extract (RAKE) algorithm to extract keywords potentially used as a “trait” or “global change driver”. We first retained the top 3,000 keywords for manual labeling. This manual labelling followed these steps:

1. Screening out irrelevant keywords (e.g., “springer”, “article”),
   1. In addition to that, we also removed keywords with a general or imprecise meaning to trait-based ecology, such as “characteristics”, “genome-wide association”, “genomic regions”, and others (Table S2).
2. Labeling if a keyword is a synonym or specific term of “trait” (referred to as “trait terms”) or global change driver (referred to as “global change terms”)
   1. We aggregate words in “keyphrases_synonyms” to avoid counting terms with a similar meaning or exactly the same word, but written in singular or plural (e.g., biofuel and biofuels). Other examples include the keywords “cancer”, “cancer cells” and “cancer progression”, that were synonymized as “cancer” (Table S2).

**Table S2.** List of terms and manual categorization (file attached in the system).

**Table S3.** Correlation of the Procrustes rotation comparing the scores of the Principal Coordinate Analysis comparing term usage in the different disciplines / subdisciplines.

| **Terms** | **Global change drivers** | **Abiotic traits** |
| --- | --- | --- |
| Abiotic traits | 0.921 |  |
| Biotic traits | 0.942 | 0.915 |

**
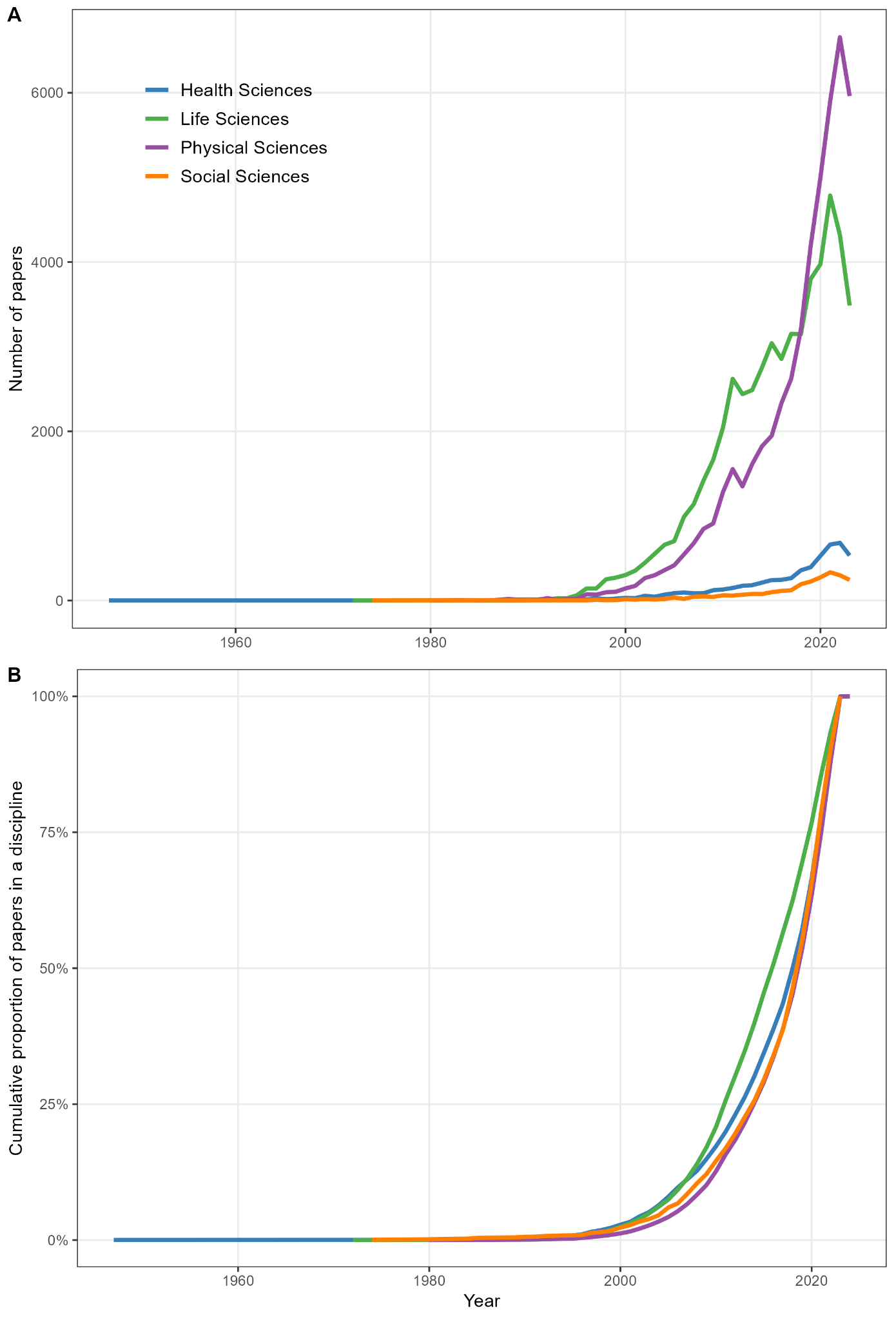
**

**Figure S1. (**A) Number and (B) cumulative proportion of scholarly publications from different science disciplines that include keywords related with traits (and similar terms) and global change drivers.

**
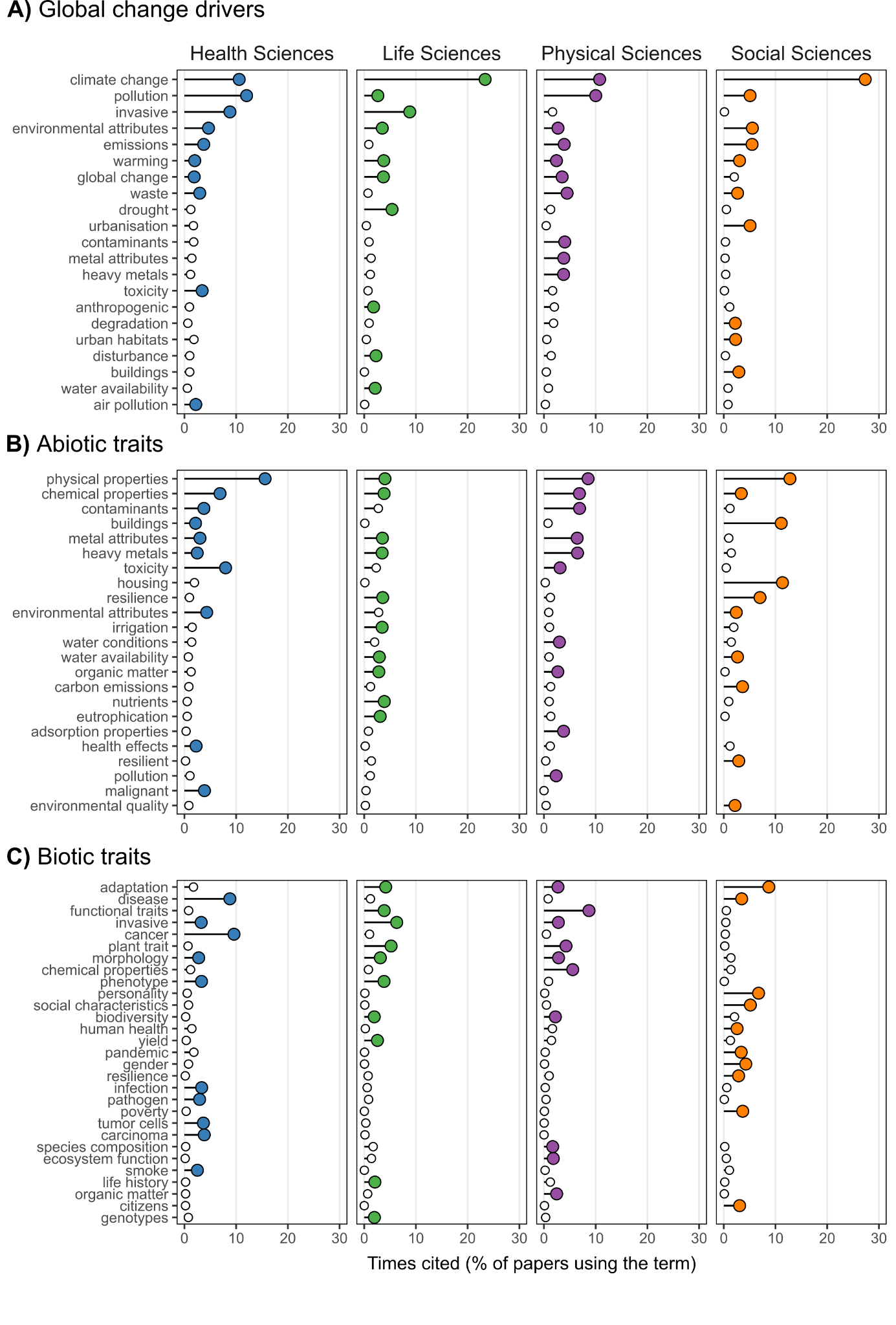
Figure S2.** The most common terms used in the four science disciplines. A) global change drivers, B) abiotic traits, and C) biotic traits. Filled circles represent the top 10 most cited terms in a given discipline, while non-filled circles represent terms that do exist in a given discipline, but are not the most common. The terms in the y-axis were organized (from top to bottom) in descending order based on the average number of papers across disciplines.
